# Ayurveda management of pulmonary mycosis: an integrative approach: a case report

**DOI:** 10.1186/s13256-022-03736-6

**Published:** 2023-02-08

**Authors:** Prasan Shankar, Bhavya Vijay, Narendra Pendse, Mahima Rahman, Vasudevan Nampoothiri

**Affiliations:** 1 Rasayana Tantra Unit, IAIM Healthcare Center, The University of Trans-Disciplinary Health Sciences and Technology, Bangalore, India; 2 Center for Clinical Research and Education, The University of Trans-Disciplinary Health Sciences and Technology, Bangalore, India; 3 Kayachikitsa Unit, IAIM Healthcare Center, The University of Trans-Disciplinary Health Sciences and Technology, Bangalore, India

**Keywords:** Pulmonary mucormycosis, Ayurveda treatment, Integrative approach, *Shvaasa roga*, Fungal lung infections, Case study

## Abstract

**Background:**

Pulmonary mycosis is a fungal infection of the lung. Antifungal treatments are used in conventional treatments; however, incomplete response and toxicity are major challenges of antifungal therapies. In Ayurveda, pulmonary mycosis is diagnosed and treated based on principles of respiratory disorders (referred to as *Shvaas Roga*) with promising outcomes.

**Case presentation:**

A > 60-year-old South Indian male patient visited Institute of Ayurveda and Integrative Medicine with complaints of cough, breathlessness, pedal edema, weight loss, uncontrolled diabetes, and anemia. Following chest X-ray, high-resolution computed tomography (HRCT) and bronchoscopy, the patient was diagnosed with a case of pulmonary mucormycosis. The patient had availed conventional allopathic treatment for 3 months including standard antifungal medication for 3 weeks. However, due to unresolved and persistent symptoms, the patient sought Ayurveda treatment. The patient was diagnosed and treated for 6 weeks as a case of *Shvaasa Roga*, a subcategory of the respiratory disorder according to Ayurveda, and was cured of the infection following an integrative Ayurveda management regime which included internal medicines, panchakarma, necessary poorvakarmas (like abhyanga and swedhana), diet and lifestyle advice, yoga and acupuncture.

**Conclusions:**

The patient was cured of fungal lung infection in 6 weeks using an integrative approach. Primary Ayurveda treatment supported with diet and lifestyle modifications, yoga, and acupuncture helped the patient to recover from illness. The patient is alive and free of disease for more than one year to date.

**Supplementary Information:**

The online version contains supplementary material available at 10.1186/s13256-022-03736-6.

## Background

Pulmonary mycoses are fungal infections of the respiratory tract that occur commonly due to the depletion of beneficial bacterial flora, inhalation of fungal spores, or dissemination of fungal infection from other infected organs in immunocompromised individuals treated with immunosuppressive drugs [1]. Pulmonary mycoses are rare opportunistic fungal infections with mortality as high as 45% as per 2021 reports [2]. In India, a cumulative burden of infection is between 137,807 and 208,177 cases, where the rate of mortality is 38.2% per annum. Diabetes mellitus and immunocompromised conditions host higher risk of infection [3]. Amphotericin B still is the first choice in the treatment of fungal infections of lungs [4]. While the emergence of newer choices of antifungals such as echinocandin antifungal agents, lipid-based drugs (such as amphotericin B) have given more flexibility, the inadequate dosing and incomplete response often leads to side effects like nephrotoxicity. Additionally, accessibility and high cost are serious challenges for patients seeking treatment in developing countries [5]. Recently, increased incidence of secondary pulmonary fungal infections has been observed in patients suffering from COVID-19 [6]. With incomplete response to standard antifungal therapies, it is important to identify and implement feasible and long-term integrative treatment choices [7–10].

Alternatively, according to the Ayurveda classification of diseases (ACD), pulmonary mycosis is diagnosed and treated based on principles of respiratory disorders (referred to as *Shvaasa Roga*). Management algorithms of *Shvaasa roga* broadly includes medicines and practices to bring back body homeostasis (using dosha-pacifying interventions), and adoption of elimination procedures (referred to as shodhana) [11]. It also gives a clear directive to physicians to ensure that food, medicine and therapies exhibit properties, which improve digestion and metabolism (deepaniya), and improve and restore balance of normal physiology [12]. Further, external therapies like oil massages and fomentation techniques, and rejuvenation procedures (referred to as rasayana) are also part of Ayurveda treatment. This principle is suitably adopted, modified as per response patterns and tailored, based on the presenting symptoms, stage of disease, strength, dominant dosha (individual’s phenotypic traits) of “the patient in consideration.” Several pulmonary infections, such as pulmonary tuberculosis, bronchiolitis, interstitial lung diseases, chronic obstructive pulmonary disease (COPD), COVID-19 have been effectively treated using Ayurveda [13–18]. With the recent large outbreak of pulmonary mucormycosis post COVID-19 infection, several published studies have expatiated the principle of mycosis management using Ayurveda [19]. Ayurveda treatment of COVID-19 associated chronic conditions were successfully applied in some cases [20–22].

In this case report, we present a case of pulmonary mycosis treated using Ayurveda in integration with yoga, acupuncture, diet and lifestyle changes. Currently, with the outbreak of COVID-19, the pulmonary fungal infection rates have increased drastically. There is a pressing need for efficient treatment and we have come across the realization that integrating medical approaches is the solution. This case report is one such example of an integrative approach where we used Ayurveda as primary treatment alongside integration with diet, yoga and acupuncture therapies for the effective management of pulmonary mycosis.

## Case presentation

The case illustrates the potential of Ayurveda theory and practice to effectively manage pulmonary mycosis.

### Chief complaints

A > 60-year-old South Indian male asthenic built patient, non-smoker presented to the outpatient department (OPD) of our healthcare center, with an ill-looking appearance. Observations showed cachexia, labored breathing, pale complexion, forward stooped body posture, whitish discoloration of the tongue, swelling in feet, flaking skin with blackish discolorations on palm and soles, and a foul intolerable odor from the oral cavity of the patient.

### Diagnosis and treatment history

Three months before initiating Ayurveda treatment, the patient was diagnosed with dengue and was treated at a conventional allopathic hospital. The patient provided no history of diabetes, and neither did his previous investigations revealed diabetes. However, during hospitalization, the patient was detected to have uncontrolled diabetes. The patient had no history of prior respiratory illness but developed a cough and expectoration with breathlessness, which got progressively worse. Systematic physical examinations and diagnostic tests like chest X-ray, electrocardiogram (ECG), high-resolution computed tomography (HRCT), endobronchial ultrasound (EBUS) aspirate, and histopathological examination (HPE) of the endobronchial biopsy revealed pulmonary mycosis.

**Fig. 1:**
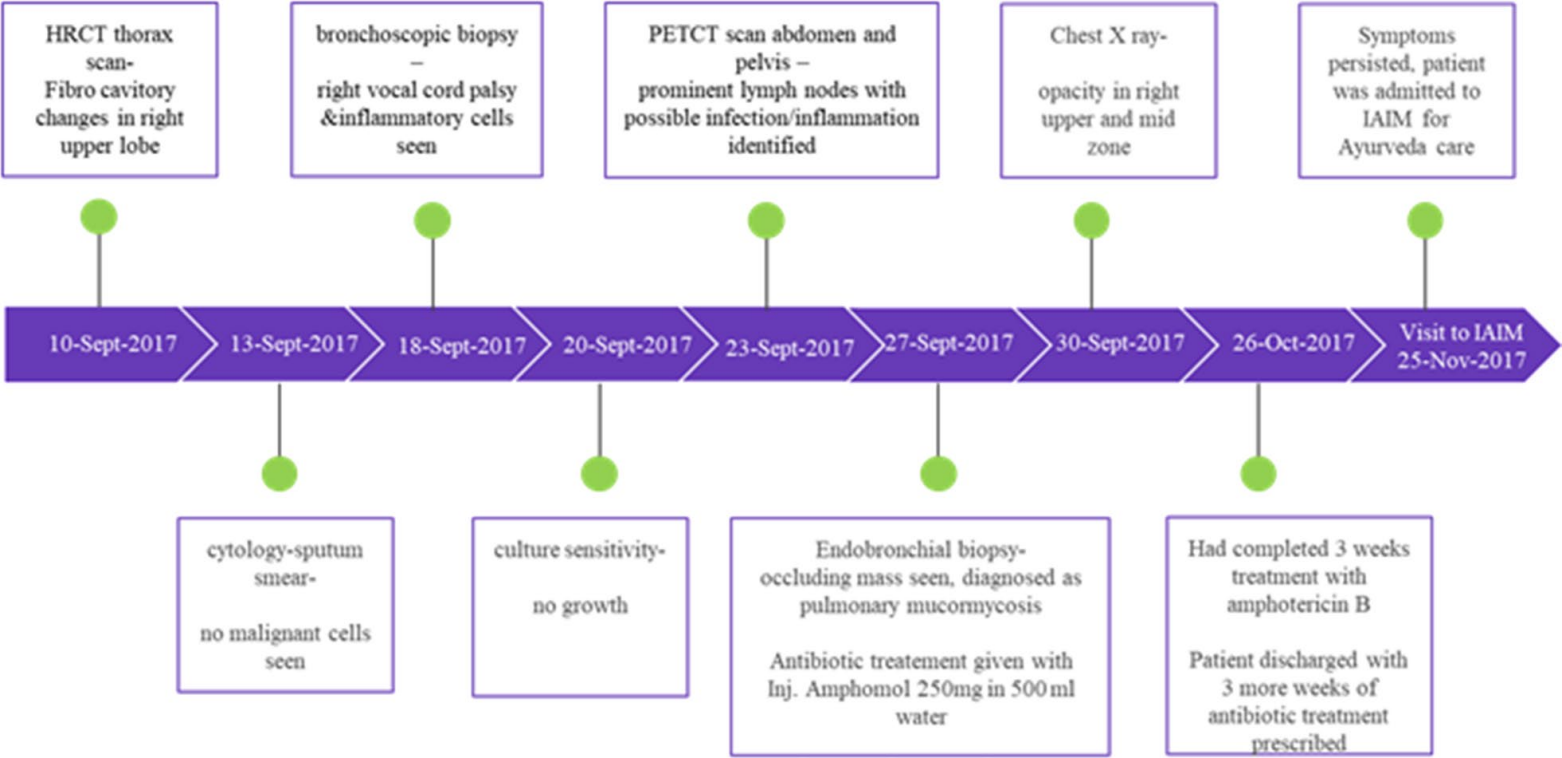
Timeline of patient’s investigations for treatment

The patient was prescribed conventional treatment with amphotericin B (twice a day), asthalin 100 inhaler SOS, and insulin human –mixtard 10 units (twice a day). Additionally, the patient was under intravenous hydration, oxygen, and nebulization periodically. However, the cough and associated symptoms worsened and the patient lost nearly 20 kgs of weight from the time of treatment initiation. About three months after non-responsiveness to conventional therapy, the patient approached our healthcare center for further management. The timeline of the patient's clinical investigations is shown in Fig. 1.

### Diagnostic assessments

Physical examination indicated increased respiratory rate, greater effort at breathing with accessory muscle usage, and bilateral crepitations. Primary observations of breathlessness, cough, and difficulty in lying down flat were associated with the ACD diagnosis of *Shvaasa roga.* Previously done culture and laboratory reports of the patient depicting lung infection, primary investigations, such as total blood count, glycosylated hemoglobin (HbA1C), hemoglobin (Hb), erythrocyte sedimentation rate (ESR), SpO2, and pulse were evaluated. A confirmatory evaluation of lung X-ray was performed to revalidate the presence of fungal infection. X-ray report revealed poor inspiratory signatures with an opaque film on the right upper and mid-zone of the lungs, which indicated microbial growth. Fibro-cavitary changes on the right upper lobe were observed in the HRCT thorax scan. Bronchoscopy evaluation revealed that the right upper lobe, the anterior segment was occluded by a lesion. Endobronchial biopsy revealed acute inflammatory exudate containing fungal filaments. The diagnosis revealed pulmonary mycosis infection.

**Fig. 2:**
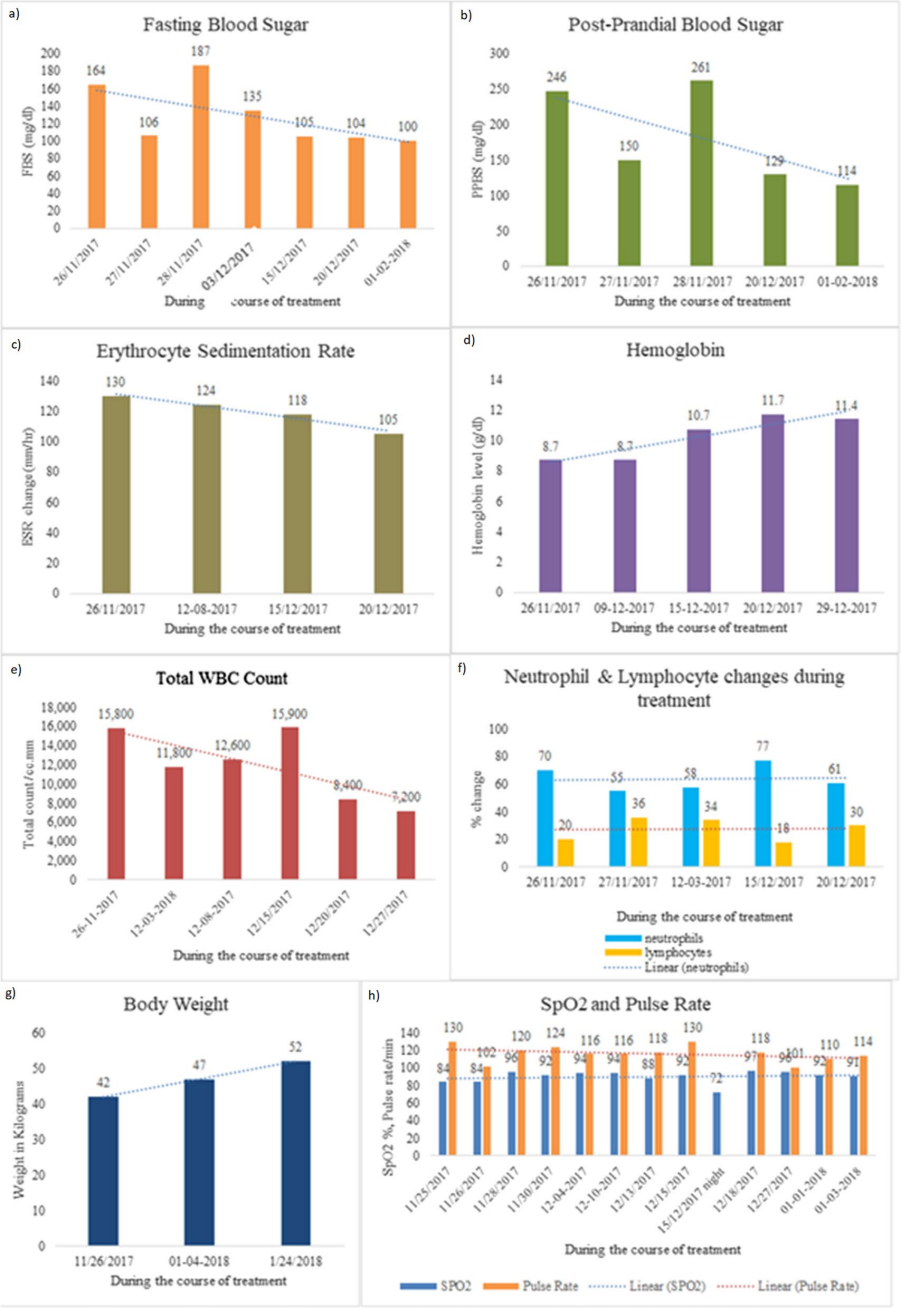
Blood parameter changes during the course of treatment. **a** Fasting blood sugar, **b** post-prandial blood sugar, **c** erythrocyte sedimentation rate, **d** Hemoglobin, **e** total WBC count, **f** neutrophil & lymphocyte changes, **g** Body weight and **h** Oxygen Saturation (SpO2) and pulse rate

As per ACD, the patient expressed traits of physical weakness along with breathlessness and was diagnosed as Vata dominant *Shvaasa Roga*. The biochemical tests were repeated at regular intervals during the treatment (Figs. 2).

**Fig. 3:**
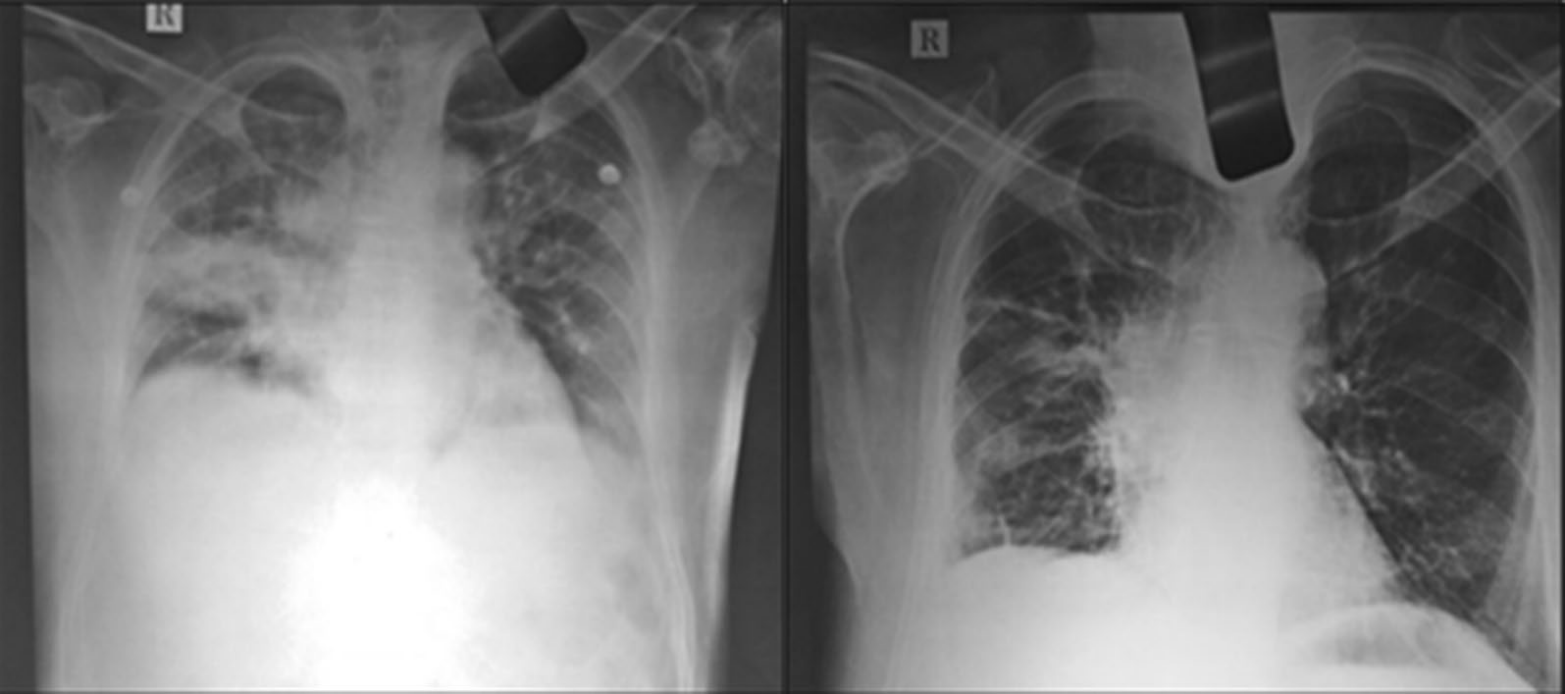
**a** X-ray of the lungs before treatment (Left Image) **b** X-ray of the lungs after treatment (Right image)

### Diagnostic challenges

As per the ACD, there is no direct correlation to fungal infections. The diagnosis is then based on etio-pathological interpretation based on treatment principles in classical Ayurveda literature. The other challenges faced were that biopsies were not performed at baseline to avoid invasive procedures. Previously obtained biopsy reports were considered for the baseline assessment of the infection. The patient was also financially weak and hesitant to repeat diagnostic tests. Due to this, the patient was treated based on symptoms, lab values, and physical examinations.

### Therapeutic interventions

After a detailed examination of the conditions, the patient's family was informed about the poor prognosis, and was admitted after obtaining high-risk consent. The patient was administered Ayurveda treatment based on the ACD classification of his condition. Considering that the patient was physically weak and Vata dominant, a dosha-pacifying line of management was adopted which included a combination of internal medications and external therapies.

Ayurveda is a clinical science that gives signs and symptoms of various vyaddhis (Diseases), and the selection of medications was made as per the expressed signs. The internal formulations used were prepared fresh daily and given to the patient [Viz *Dasamoolakaduthryadi kashaya, Indukantham Kashaya, Punarnavadi kashaya, Fresh juice of Tulsi (Ocimum sanctum), Vidaryadi kashaya, Pippali (Piper longum)*]. Other formulations were procured from Good Manufacturing Practices (GMP)-certified pharmaceutical manufacturers [including *Vaishwanara Churna, Swasanada Gulika, Agastya Rasayana, Sitopaladi Churna, Vilvadi Lehya, Vidaryadi Ghrita*]. All of these formulations are listed in the Ayurveda Pharmacopoeia of India (API). These formulations were administered at different timelines during the patient's journey toward recovery (See Additional file 1: Table S1).

External therapies such as warm oil application (abhyanga) to the chest and back region, followed by warm fomentation (nadi sweda) procedures were administered daily from day 1. Non-pharmacological interventions such as yoga and Acupuncture therapies were introduced after week 3 of the treatment protocol.

Diet as per the patient’s need was carefully designed during the 6 weeks of hospital stay. A light, easily digestible diet was recommended to the patient. It was observed that the patient consumed food in small quantities every 2–3 h. The patient’s symptoms were evaluated regularly, and the treatment was modified accordingly. Further, during treatment, to improve and regain strength, physiological function, and rehabilitation of the respiratory system, yoga, pranayama and acupuncture were advised from day 24 onwards till the end of the treatment (Additional file 1: Table S2). The sessions of yoga and acupuncture were customized to the patient's condition. In particular, treatment was provided to improve the lung channels (acupoints L1,7,9,11), enhance immunity (points LI4,11), promote blood and capillary circulation (SL6, SP9,10,36) and overall health (points CV4,6,9,17,GV20) [23, 24].

The laboratory investigations were evaluated at frequent intervals during the treatment.

### Follow-up and outcomes

The clinical symptoms gradually resolved at the end of week-6 of the intervention. The added breath sounds markedly decreased, pedal edema decreased with a reduction in added respiratory sounds. The inspiratory breath sounds improved, which indicated improved lung function. The patient reported an improvement in appetite by week 3. By week 4, the patient had a strong desire to eat normal food. Subsequently, improved strength, better complexion, reduction in foul odor, and better digestion and metabolism were observed. From week 5 onwards, till his discharge, the focus was mainly on tissue nourishing interventions.

Further, from the laboratory findings, fasting blood sugar (FBS), and post prandial blood sugar (PPBS) levels were normalized, and the patient gained weight as shown in Fig. 2a, b and g. The hemoglobin levels (Hb) increased gradually, and total blood count (TC), neutrophils, and ESR levels were back to normal range, see Fig. 2c–f. Pulse rate and SpO2 were also stabilized at the end of the treatment as shown in Fig. 2h. Finally, a confirmatory chest X-ray during the first follow-up revealed no pulmonary opacity as shown in Fig. 3a, b.

The patient was followed up periodically after 2 weeks, 4 weeks and 1 year of treatment to assess recurrence (Additional file 1: Table S3). The patient continued to have normal breathing patterns with no infection recurrence. The patient is currently doing well.

### Adverse event

During the in-patient treatment, one adverse event was reported between in week 3 (from day 21 to day 24). The patient had frequent liquid stools along with abdominal pain and an episode of severe cough, and blackish-green vomiting at night, the blood oxygen saturation level dropped to 85% during which the patient was provided with oxygen and nebulization. The cause of the event was identified to be a deviation from the recommended diet. All the treatment interventions were stopped and the patient was given Ayurvedic antidiarrheal medicines (Mebarid [25] and Dadimashtaka churna [26]). Finally, as the patient's symptoms reduced, the standard Ayurveda treatments were resumed.

## Discussion

Pulmonary mycosis is rare opportunistic infection, and clinical studies on Ayurveda management of pulmonary mycosis are not reported yet. Many preclinical studies have reported the possible action of Ayurveda treatment of fungal pathogens and infections. Antifungal activity of herb extracts *Allium sativum, Zingiber officinale, Ocimum sanctum, Curcuma longa, Piper betel* [27], herbal formulations like *Asanadi Kwatha Choorna* on *Mucor* species have reported effective prevention of *Mucor* species [28]. Similarly, *gandhaka rasayana* used against 3-bacterial and 4-fungal species, with Fluconazole as standard antimicrobial, showed effective outcomes in inhibiting the fungal and bacterial growth against fungal species *Trichophytum rubrum* and *Aspergillus niger* [29]. Similar preclinical studies on various formulations against fungal species have been done [30–32]. However, in the clinical setting, the pathogenesis and the synergistic effects of the formulations play a key role. In addition, the dose and the practical management of invasive fungal infections need more attention, unlike in a controlled preclinical environment. Some clinical studies in Ayurveda have demonstrated the capacity of its systemic diagnosis and treatments for various bacterial, viral, fungal, and parasitic infections [33–37]. In this case, the Ayurveda clinical management of pulmonary mucormycosis is reported for the first time.

### Ayurveda classification of *Shvaasa roga*

**Fig. 4:**
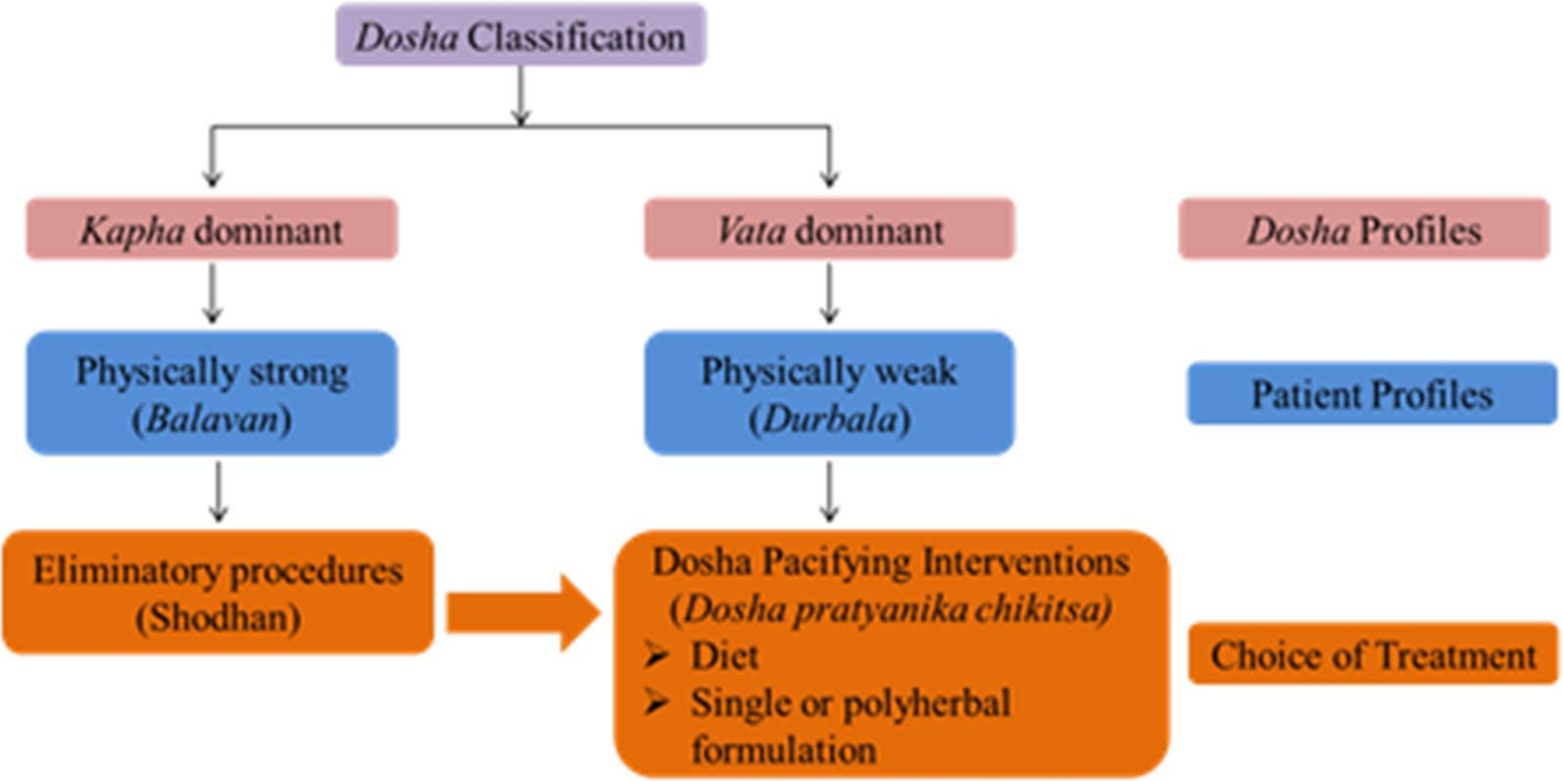
Schematic representation of diagnostic and treatment algorithm

According to the ACD, pulmonary mycosis is diagnosed and treated based on principles of respiratory disorders (referred to as Sadhyasadhyata) of *Shvaasa roga* [38]. In the Chapter about respiratory disorders in the Caraka Samhita, one of the foundational texts of Ayurveda, a detailed description of etiology (referred to as Nidana), symptomatology (referred to as Lakshana), pathogenesis (referred to as Samprapti), classification, management strategies (referred to as Chikitsa) and prognosis (Referred to as Sadhyasadhyata) of *Shvaasa roga*, has been described [38]. Patients with *Shvaasa roga* are classified within the framework of Ayurveda’s Humoral theory viz dosha of presentation into Vata dominant or Kapha dominant [39]. This classification aids in choosing the appropriate line of management (Fig. 4).

Management algorithms of *Shavaasa roga* broadly include dosha-pacifying interventions (referred to as dosha pratyanika chikitsa), adoption of elimination procedures (referred to as shodhana) indicated by Charaka. The principle of management of respiratory disorders gives a clear directive to physicians to ensure that food, medicine and therapies should be Vata and kapha pacifying in nature, exhibiting properties, which improve digestion and metabolism (Deepaniya), and improve and restore balance of normal physiology of Vata. In addition, external therapies viz oil massages and fomentation techniques, and rejuvenation procedures (referred to as rasayana) are also part of this algorithm.

Therefore, the patient was treated based on the guidelines detailed in the chapter of *Shvaasa roga*. Etiopathological relevance of *Shvaasa roga* indicates Pranavaha srotas, Annavaha Srotas and Udaka vaha srotas are primarily involved [40]. The clinical presentation of the patient also concur with this description. The patient exhibited symptoms such as breathlessness, cough, difficulty in lying down flat (Shvaasa, Kasa, Asinolbhate soukhyam of Shvaasa roga). Further, discoloration and foul smell are seen in presentations of subcategories of *Shvaasa roga* such has Maha Shvaasa. Shvaasa is primarily a Vata-kaphaja presentation. The chikitsa sutra of Shvaasa implies that food, medicine and therapies should be Vata-kapha pacifying in nature, should be hot in potency, and should improve and restore balance of normal functions of Vata [39]. The line of treatment followed was correcting metabolism (Agni), symptomatic management of patient complaints (Vyadhipratyanika chikitsa), rejuvenation (Rasayana chikitsa), and preventing relapse (Apunarbhava chikitsa).

### Intervention

In this case study, in the first week of treatment, the patient was treated to alleviate the infection and associated symptoms of breathing difficulties and cough, as well as diabetes. *Dashamoola* is a combination of 10 herbs reported to possess anti-analgesic and anti-inflammatory effects, it decreased edema, and improved digestive integrity and rigor [41]. In addition, hingula (cinnabar), pippali (*Piper longum*), dalchini (*Cinnamomum camphora*) are known to augment immune responses and act as emulsifying agents, improve capillary blood circulation and increase gut functioning. Further, pippali (*Piper longum*), Tulsi [*Ocimum sanctum*], ginger [*Zingiber officinale*][42–44], parnayavani [*Coleus aromaticus*], and honey, exhibit anti-tussive, anti-inflammatory and antioxidant potential that rapidly alleviate discomforts of cough, phlegm and breathlessness. Alongside, these herbs *Indukantham kashayam, vaiswanara choornam, vilawadi lehyam, sitopaladi choornam,* have specific classical indications in Ayurveda literature, for enhancing metabolism and for use in respiratory conditions were administered. To improve nourishment and immunity, the patient was prescribed goat’s milk in addition to the herbal medications. Goat milk is reported to inhibit the growth of fungal spores that cause lung infections. Additionally, goat milk nourishes the body, aids in rehydration, gastrointestinal protection, and exhibits antifungal and antimicrobial properties [45, 46].

Further, Diabetes mellitus and hyperglycemia reduced immune responses, stimulating fungal proliferation [5]. Many studies show that Diabetes mellitus may affect the pharmacokinetics and pharmacodynamics of various drugs including antifungals, causing slower absorption and reduced susceptibility to antifungals [47–49].The improvement in blood glucose parameters observed in this case indicates that the Ayurveda management system not only works against infection, but exerts a more systemic effect that restores physiological balances and resilience [50, 51].

### Diet management

The insight from the classical literature of Ayurveda points out that respiratory disorders originate in the stomach and gut [38]. Emerging evidence has unraveled the link between gut microbiota and lung mycobiome. Gut microbiota and lung mycobiome have shown to influence one another and play a key role in regulating inflammation, homeostasis, and immune response of the respiratory system and could be an important area for future research [52]. Therefore, focusing on improving gut health and metabolism plays a key role in the overall restoration of respiratory health. Therefore, correcting the impairments in digestive processes (a physiological concept referred to as Agni in Ayurveda) is the primary step in the management. In this case, the patient did not undergo the classical process of shodhana as the patient had poor strength (Bala). Nonetheless, diet patterns were followed based on the principle of Samsarjana Krama (Sequential administration of liquid diet to a normal diet to kindle the Agni or digestive processes) [53]. Changes in treatment strategy were guided by careful observation of clinical parameters of metabolism such as digestive ability (agni), strength (Bala), appearance (varna), odor (Gandha), and careful observation of the dynamic changes in the clinical stages (Avastha).

### External therapies

Oil massages and steam is the first step in the management of *Shvaasa roga*. It regulates sensory and tissue stimulations, which results in vasodilation and helps in easy expectoration of sputum which is sticky, causes blockage of ventilation, and reduces the inhalation capacity of the lungs^42^. In addition, non-pharmacological interventions such as yoga and acupuncture therapies were also prescribed to improve overall respiratory function [54, 55]. Yoga and Acupuncture regimens suitable and tailored to the individual were advised. Yoga sessions including bedside pranayama practices, and movements improving chest expansion, sukshma vyayama (gentle warm-up practices coordinated with breathing) was done to loosen the muscles by stretching and bending, then, sukshma kriya and pranayama were done to cleanse out the toxins from the respiratory channels [56]. Acupuncture was rendered by a registered medical practitioner who also specializes in acupuncture. as to improve the impulses and efficiency of the lung channels. Acupuncture points were chosen to improve the lung channels (L1,7,9,11), enhance the immunity (LI4,11), blood and capillary circulation (SL6, SP9,10,36) and overall governance (CV4,6,9,17,GV20) [23, 24].

By the end of treatment, the patient had betterment in appetite and reduced breathing difficulties. The patient was physically active and enthusiastic, and gained body weight as well. The patient was discharged by the end of sixth week and was advised to continue the medications to maintain and prevent the infection recurrence. The patient had no recurrence of infection and reported with no complaints during the follow-up visits.

This study is the first paper evidencing the potential of Ayurveda treatment in fungal respiratory infection treatment.

### Value of integrative approach

The treatment of pulmonary fungal infections is challenging. Firstly, concerning the diagnostic and prognostic difficulties. Secondly, limited treatment options with poor outcomes and high fatality rates of the disease. Further, with the rapid rise of recent unanticipated black fungus infections post COVID-19, the fatality rates of fungal infections are getting higher. Lastly, the overuse of steroids and drugs resulting in irreversible side effects is another concern in the treatment of fungal infections. It is important to identify and utilize the diagnostic and treatment modalities for the early detection and treatment of these diseases. A rational application of multidimensional integrative treatments could aid in optimal treatment in challenging conditions such as pulmonary mycoses.

In this case study, we utilized the diagnostic tools for the identification and assessment of disease signs and symptoms. We implemented the holistic therapeutic potential of Ayurveda interventions in the management and rectification of infection and restoring normal physiology including blood sugar control. Further, with the help of diet and lifestyle advice, we normalized the metabolic functionalities. Comprehensive integration of yoga, exercise, massage, and acupuncture helped improve the breathing capacity, and strength and immunity to aid the patient to regain physical strength, restoring lung functionality and resilience.

While in the conventional approach (allopathy), treatment strategies remain more or less standard (antifungal treatments), this case report has used a combination of treatments from various disciplines. Ayurveda’s patient-centric integrative approach makes changes, according to the patient’s response, adapts, and makes alterations in the intervention strategy with the changing clinical status. This pattern is more personalized because it is inherently adaptive and depends on an individual's systemic immune response.

### Limitations of the study

While a case report has the advantage of describing the complete diagnostic and treatment approaches in enormous detail, this report is limited by the fact that it is a single case. While Ayurveda was primarily used in the treatment, some integrative treatment methods were incorporated for emergency medicine. Along with Ayurveda diagnosis and observations, standard biochemical blood tests and as well as symptomatic interventions has been described. It was therefore clear that an integrative approach was better suited to manage the case comprehensively.

## Conclusion

In this case study, an integrative approach of management was used for the treatment of pulmonary mucormycosis. Ayurveda being the primary line of treatment, in addition to Yoga, Acupuncture, diet, exercise and lifestyle advice were used in effectively treating the infection in 6 weeks of time.

## Supplementary Information

**Additional file 1: Table S1. **Rationale behind the choice of medicines. **Table S2. **Treatment chart. **Table S3. **Follow-up treatment chart.

## Data Availability

Not applicable.

## Abbreviations

ACD: Ayurveda classification of diseases
API: Ayurveda Pharmacopoeia of India
EBUS: Endobronchial ultrasound
ECG: Electrocardiogram
ESR: Erythrocyte sedimentation rate
FBS: Fasting blood sugar
GMP: Good manufacturing practices
Hb: Hemoglobin
HbA1C: Glycosylated hemoglobin
HPA: Histoplasmosis
HPE: Histopathological examination
HRCT: High-resolution computed tomography
OPD: Outpatient department
PPBS: Post prandial blood sugar
TC: Total blood count
