# Supplementary material for: Ayurveda management of pulmonary mycosis: an integrative approach: a case report
Source: J Med Case Rep. 2023 Feb 8;17:48. doi: 10.1186/s13256-022-03736-6 (PMC9906598; doi:10.1186/s13256-022-03736-6)
Supplement: Supplementary file 1 — Additional file 1: Table S1. Rationale behind the choice of medicines. Table S2. Treatment chart. Table S3. Follow-up treatment chart. [file 13256_2022_3736_MOESM1_ESM.docx]

**Additional file 1**

**Table S1: Rationale behind the choice of medicines**

| **Sl. no.** | **Medicine name** | **Action on** | **Nature and function** | **Targeted on** | **Effect** |
| --- | --- | --- | --- | --- | --- |
| 1 | Vaishwanara churna | Kapha vata shamak | Rochana (Improves taste), Deepana  (Digestive), Pachana (carminative),  Vatanulomana (Normalizes downward movement of vata) | Annavaha srotas | Pachana |
| 2 | Dasamoolakatutryadi | Vata kapaha hara, | Pachana (Carminative), Shotha hara (Reduces edema), Shvaasa kasa hara (Alleviates Respiratory dyspnea/ cough) | Annavaha srotas, Rasavaha srotas, Pranavaha srotas | Deepana |
| 3 | Indukanta kshaya | Vata hara, | Deepana (Digestive), Vata hara (alleviates vata), Srotoshodhana (improves microcirculation), Anulomana (Normalizes downward movement of vata), Rasayana (Rejuvinative), Shvaasa kara hara (Alleviates Respiratory dyspnea/ cough) | Annavaha srotas, Rasavaha srotas | Shamana |
| 4 | Tulsi [*Ocimum sanctum*], ginger [*Zingiber officinale*] parnayavani [*Coleus aromaticus*] | Vata Kapha hara | Deepana (Digestive), Shvaasa kasa hara (Alleviates Respiratory dyspnea/ cough) | Annavaha-amashaya, Pranavaha,  Rasavaha, Medovaha, Shukra vaha | Shamana |
| 5 | Punarnavadi Kashya | Kapha hara | Shotha hara (Reduces edema), Kleda shoshana (regulates fluid balance), Shvaasa, kasa hara (alleviates Respiratory dyspnea/ cough) | Rasa, Mutravahasrotasa, Udakavahasrotas | Shamana |
| 6 | Rasa sindura | Vata kapha hara, | Pachana (Carminative), Deepana (Digestive), Dhatvagni and bhutagni balya (improves tissue metabolism), Sroto shodhaka (improves microcirculation), Ojus vardhana (improves Vitality), Shvaasa kasa hara (Alleviates Respiratory dyspnea/ cough) | Rasavaha srotasa, Pranavaha srotas, Majja vaha shukra, Shukra vaha shukra | Rasayana |
| 7 | Swasananda Guilika | Vata-kapha, | Deepana (Digestive), Ushna (Endothermic), Dhatvagni and bhutagni balya (improves tissue metabolism), Sroto shodhaka (improves microcirculation), Shvaasa kasa hara (Alleviates Respiratory dyspnea/ cough) | Pranahava srotas | Rasayana |
| 8 | Sitopaladi churna | Kapha-pitta hara | Deepana (Digestive), Shotha hara (Reduces edema), Sroto shodhana (improves microcirculation), Rochana (Improves taste), Rasa Dhatu kara (Nourishes Rasa Dhatu), Ojus vardhana (improves vitality), Rasayana (rejuvenative), Shvaasa kasa hara (Alleviates Respiratory dyspnea/ cough) | Rasavaha srotas, Pranavaha srotas | Rasayana |
| 9 | Bilvadi Leyha | Kapha-Vata Hara | Deepana (Digestive), Stambhana (Anti diarrheal/emetic), Chardi hara (Antiemetic), Ruchi (improves taste), Rasayana (Rejuvinative), Shvaasa kasa hara (Alleviates Respiratory dyspnea/ cough) | Annavaha srotas, Rasavaha srotas | Rasayana |
| 10 | Vidaryadi Kashya | Vata hara | Hridya (Cardio tonic), Hridya (Cardiac Tonic), Rasayana (Rejuvinative), Shvaasa kasa hara (Alleviates Respiratory dyspnea/ cough) | Pranavaha srotas, Rasasrotas, Shukra  Ojus | Rasayana |
| 11 | Pippali | Kapha-Vata hara | Pachana (Carminative), Deepana  (Digestive), Anulomana (Normalizes downward movement of vata), Kapha hara (alleviates kapha dosha), Rasayana (Rejuvenative), Shvaasa kasa hara (Alleviates Respiratory dyspnea/ cough) | Pranavaha srotas, Rasasrotas | Rasayana |
| 12 | Indukanta ghrita | Vata hara, | Deepana (Digestive), Vata hara (alleviates Vata dosha), Srotoshodhana (improves microcirculation), Anulomana (Normalizes downward movement of vata), Rasayana (Rejuvenative), Shvaasa kasa hara (Alleviates Respiratory dyspnea/ cough) | Pranavaha srotas, Rasasrotas | Rasayana |
| 13 | Agastya Rasayana | Kapha vata hara, | Rasayana (Rejuvenative), Srotoshodhana (improves microcirculation), Anulomana (Normalizes downward movement of vata), Shvaasa kasa hara (Alleviates Respiratory dyspnea/ cough) | Pranavaha srotas, Rasasrotas | Rasayana |
| 14 | Goats Milk | Vata Hara | Rasayana (Rejuvenative), Brimhana (improves Tissue Nourishment), Shvaasa kasa hara (Alleviates Respiratory dyspnea/ cough) | Annavahasrotas, Pranavaha srotas, Rasasrotas | Rasayana |

**Table S2: Treatment chart**

| Weeks | Symptoms | Treatment | Dosage | Co-treatment/therapy | Clinical observations |
| --- | --- | --- | --- | --- | --- |
| Week 1 | **Major symptoms** | **Standard treatment including** | **Standard dose of drugs** | **Standard therapies include** |  |
|  |  |  |  |  |  |
|  | Nausea, vomiting, cough, breathlessness fatigue, weakness, disturbed sleep at night, yellowish sputum. Other symptoms: swelling in the feet, incomplete evacuation of bowels, reduced appetite, difficulty in walking, Flaking of skin over palms and soles, blackish discoloration, foul odor | 1. Dasamoolakaduthryadi kashaya, | 20 mL-0-20 mL, (6 AM and 6 PM) | Insulin injection 10-0-10 units | Cough aggravated at night at the start, gradually decreased by day 4. Day sleep was improved by day 5. Nausea was reduced by day 6. Other symptoms persisted |
|  |  |  |  |  |  |
|  |  | 2. Indukantham Kashaya | 20 mL-0-20 mL, (6 AM and 6 PM) | Steam inhalation once a day |  |
|  |  |  |  |  |  |
|  |  | 3. Swasanandam Gulika | 1-1-1, before food | Oil application (*Abhyanga)*+ Steam *(Nadi sweda),* twice daily |  |
|  |  |  |  |  |  |
|  |  | 4. Vaiswanara Choornam | 1 tsp at 11 AM with warm water |  |  |
|  |  |  |  |  |  |
|  |  | 5. Vilwadi Lehyam | 2 tsf-0-2 tsf, before food |  |  |
|  |  |  |  |  |  |
|  |  | 6. Dasamoola paneeya | 1 L/day |  |  |
|  |  |  |  |  |  |
|  |  | 7. Sitopaladi choornam in combination with Chousistapipli Powder, Karpooradi churna and Shataputi Abhraka bhasma | 1 tsf with honey, 1-1-1 after food |  |  |
|  |  |  |  |  |  |
|  |  | 8. Combination of *Ocimum tenuiflorum* (Tulasi), *Plectranthus amboinicus* (Parnayavani), *Zingiber officinale* (Shunti Svarasa) | 5 mL with honey, twice a day after food |  |  |
|  |  |  |  |  |  |
|  |  | Diet: Mainly Medicated rice gruel (Panchakola Yavagu), Parched rice water (Laja Peya), Finger millet Semoline gruel | | |  |
|  |  |  |  |  |  |
| Week 2 | Expectoration increased, feet swelling persists, tiredness was seen, Appetite continues to be reduced, Fatigue persist, disturbed sleep during night |  |  |  |  |
|  |  | 1. In continuation with treatments from week 1, |  | In continuation with therapies from week 1, | Expectoration and cough reduced by day 9, Nausea reduced further; night sleep improved by day 10, all the symptoms reduced and there was an overall betterment by day 13 |
|  |  |  |  |  |  |
|  |  | 2. Dasamoola katutreyam kashayam (fresh) | 30 mL thrice daily B/F | Steam inhalation with Pranadhara drops was given |  |
|  |  |  |  |  |  |
|  |  | 3. Punarnavadi kashayam | 30 mL thrice daily B/F |  |  |
|  |  |  |  |  |  |
|  |  | 4. Swasanandam Gulika | 1-1-1, before food |  |  |
|  |  |  |  |  |  |
|  |  | 5. Vaiswanara Choornam | 1 tsp at 11 AM with warm water |  |  |
|  |  |  |  |  |  |
|  |  | 6. Vilwadi Lehyam | 2 tsf-0-2 tsf, before food |  |  |
|  |  |  |  |  |  |
|  |  | Diet: Mainly Medicated rice gruel (Panchakola Yavagu), Parched rice water (Laja Peya), Finger millet Semoline gruel | | |  |
|  |  |  |  |  |  |
| Week 3 | Mild cough | Continuation of treatments from week 1 |  | Continuation of therapies from week 1 | The patient felt better by day 16 |
|  |  |  |  |  |  |
|  |  |  |  |  |  |
|  | Adverse event: | Treatment was stopped on day 17 |  |  | Adverse event was reported due to consumption of biscuits, chats and spiced rice on the previous night. |
|  |  |  |  |  |  |
|  | Day 17 onwards, 6-7 episode of loose stools, weakness, pain in the abdomen | 1. Mebarid | 2 tablets, 1-1-1, after food |  |  |
|  |  |  |  |  |  |
|  |  | 2. Dadimashtaka churnam | ½ tsf four time with honey |  |  |
|  |  |  |  |  |  |
|  | Day 18 onwards: Patient is stable, no fresh complaints of loose stools and abdominal pain | 1. Combination of Sitopaladi (50 g), Chousistapipli powder (10 g), Karpooradi churna (10 g), Shataputi Abhraka | ½ tsf, 3 times a day | Yoga and pranayama sessions were started |  |
|  |  |  |  |  |  |
|  | Day 19 onwards, | 1. Combination of Sitopaladi (50 g), Chousistapipli powder (10 g), Karpooradi churna (10 g), Shataputi Abhraka | ½ tsf, 3 times a day |  | By day 20, Fatigue reduced, tiredness reduce, no episode of loose stool, and swelling reduced |
|  |  |  |  |  |  |
|  |  | 2. Dasamoola katutreyam kashayam (fresh) | 30 mL thrice daily B/F |  |  |
|  |  |  |  |  |  |
|  |  | 3. Punarnavadi kashayam | 30 mL, 1-1-1, before food |  |  |
|  |  |  |  |  |  |
|  |  | 4. Swasanandam Gulika | 1-1-1, before food |  |  |
|  |  |  |  |  |  |
|  |  | 5. Vaiswanara Choornam | 1 tsp at 11 AM with warm water |  |  |
|  |  |  |  |  |  |
|  |  | 6. Vilwadi Lehyam | 2 tsf-0-0 at 11 AM once daily |  |  |
|  |  |  |  |  |  |
|  |  | 7. Dasamoola paneeyam | 1 L/day |  |  |
|  |  |  |  |  |  |
|  |  | 8. Tulasi, Parnayavani, Shunti Svarasa with honey | 5 mL, twice a day A/F |  |  |
|  |  |  |  |  |  |
|  |  | Diet: Mainly Medicated rice gruel (Panchakola Yavagu), Parched rice water (Laja Peya), Finger millet Semoline gruel, rice and rasam | | |  |
|  |  |  |  |  |  |
| Week 4 | Adverse event: |  |  |  |  |
|  | Day 20, 5 episodes of loose stools at night. | Vilvadi gulika | 2 tabs, at 11 AM and 4 PM | Insulin 10-0-10 units |  |
|  |  |  |  |  |  |
|  | Day 21, Complains of throat irritation | 1. Tulasi, Parnayavani, Shunti Svarasa with honey | 5 mL, twice a day A/F | insulin was stopped  Oil massage (Abhyanga) was started from day 25 | Cough and tiredness reduced by day 21, appetite improved |
|  |  |  |  |  | Energy levels – better, |
|  |  | 2. Gargling with salt + turmeric | Twice a day | Acupuncture was started from day 24 | Lungs were clear on auscultation, |
|  |  |  |  |  |  |
|  |  | 3. Standard treatment was continued |  | At night, oxygen was given along with nebulization |  |
|  |  |  |  |  |  |
|  | Day 21 night, severe cough at night time, associated with dark green vomitus, Patient was referred to Allopathic physician | 1. Azithromycin, 250 mg, 1-0-1, for 3 days,  2. Chlorpheniramine Maleate (2 mg) + Paracetamol (500 mg) + Phenylephrine (10 mg), 1-1-1, for 3 days |  |  |  |
|  |  |  |  |  |  |
|  |  |  |  |  |  |
|  |  |  |  |  |  |
|  | Day 22, the patient was feeling weak, and fatigued | 1. Dasamoola katutreyam kashayam (fresh) | 30 mL thrice daily B/F |  | Pain in the flanks reduced by day 25 |
|  |  | 2. Punarnavadi kashayam | 30 mL thrice daily B/F |  |  |
|  |  |  |  |  |  |
|  |  | 3. Swasanandam Gulika | 1-1-1, before food |  |  |
|  |  |  |  |  |  |
|  |  | 4. Vaiswanara Choornam | 1 tsp at 11 AM with warm water |  |  |
|  |  |  |  |  |  |
|  |  | 5. Dasamoola paneeyam | 1 L/day |  |  |
|  |  |  |  |  |  |
|  |  | 6. Tulasi, Parnayavani, Shunti Svarasa with honey | 5 mL, twice a day A/F |  |  |
|  |  |  |  |  |  |
|  |  | 7. Gargling with salt + turmeric | Twice daily |  |  |
|  |  |  |  |  |  |
|  |  | 8. Goat milk + Pippali churna | 50 mL + ½ tsf Pippali churna |  |  |
|  |  |  |  |  |  |
|  |  | Diet: Mainly Medicated rice gruel (Panchakola Yavagu), Parched rice water (Laja Peya), Finger millet Semoline gruel, rice and rasam | | |  |
|  |  |  |  |  |  |
| Week 5 |  |  |  |  | Patient was comfortable, feeling better, with no fresh complaints |
|  | Complains of tiredness and Mild cough at night | Azithromycin and Chlorpheniramine Maleate (2 mg) + Paracetamol (500 mg) + Phenylephrine (10 mg) were stopped, and all other treatment from week 4 was continued |  | Oil massage (Abhyanga) until day 30 |  |
|  |  | Diet: Mainly Medicated rice gruel (Panchakola Yavagu), Parched rice water (Laja Peya), Finger millet Semoline gruel, rice and rasam | | |  |
|  |  |  |  |  |  |
| Week 6 | No symptoms, patient feels better | all the treatment from week 5 was continued |  |  | The patient’s cough has reduced, appetite improved, was able to walk, was able to lie flat on the bed, sleep quality had improved, bowels were clear everyday, was energetic |
|  |  |  |  |  |  |
|  |  |  |  |  |  |
|  |  |  |  |  |  |
|  |  |  |  |  |  |
|  |  |  |  |  |  |
|  |  |  |  |  |  |
|  |  |  |  |  |  |
| Discharge | Patient was comfortable, stable appetite, improved, was able to walk, was able to lie flat on the bed, sleep quality had improved, bowels were clear every day, was enthusiastic and was fit to discharge | Vidaryadi gritha | 2 tsf-0-2 tsf | Balashwagandha for external application |  |
|  |  |  |  |  |  |
|  |  | Vidaryadi kashaya with 40 mL hot water | 20 mL-0-0 |  |  |
|  |  |  |  |  |  |
|  |  | Vilwadi leham | 2 tsf-0-2 tsf |  |  |
|  |  |  |  |  |  |

**All the Ayurveda medicines used are approved as per the National Ayurvedic Pharmacopoeia of India (API)*

**Table S3: Follow-up treatment chart**

|  | **Follow up 1** | **Follow up 2** | **Follow up 3** |
| --- | --- | --- | --- |
| ***Symptoms*** | The patient symptoms improved, no recurrence of breathlessness seen | Patient reported improved health with alleviated breathing difficulties | Patient reported improved health with no episodes of serious cough |
| ***Internal therapies*** | Dashamoolakaduthryam, 20 mL-0-20 mL for 2 weeks  Indukantham gritha, 2 tsf-0-2 tsf for 2 weeks  Vaiswanara choornam, 1 tsf-0-1 tsf for 2 weeks  Agasthya rasayan followed by goat milk, 0-0-1 tsf for 2 weeks | Dashamoolakaduthryam, 20 mL-0-20 mL for 2 weeks  Indukantham gritha, 2 tsf-0-2 tsf for 2 weeks  Vaiswanara choornam, 1 tsf-0-1 tsf for 2 weeks  Agasthya rasayan followed by goat milk, 0-0-1 tsf for 2 weeks | Dashamoolakaduthryam, 20 mL-0-20 mL for 2 weeks  Indukantham gritha, 2 tsf-0-2 tsf for 2 weeks  Agasthya rasayan followed by goat milk, 0-0-1 tsf for 2 weeks |
| ***External therapies*** | Dhanwantharam thailam mixed with Karpooradi thailam application on chest for 14 days | Karpooradi thailam application on chest for 14 days | Dhanwantharam thailam application on chest  Udwartana sarvanga massage for 7 days  Abhyanga sarvanga massage for 7 days  Nadi sweda (steam) to chest and back for 7 days  Yoga |
